# “Frontrunners”: An Investigation of the Discursive Construction of “Women Politicians” Intersectional Identity

**DOI:** 10.5964/ejop.v15i3.1557

**Published:** 2019-09-27

**Authors:** Patrizia Milesi, Augusta Isabella Alberici

**Affiliations:** a Department of Psychology, Catholic University of the Sacred Heart, Milan, Italy; Georgetown University, Washington, DC, USA

**Keywords:** intersectional identity, politicized identity, gender discrimination in politics, women politicians, women politicians’ competence

## Abstract

This paper explores how female politicians discursively construct their intersectional identity as “women politicians.” We interviewed 10 female politicians in charge of local political offices and examined how they talked about the boundaries and contents of their “women politicians” identity. When talking about identity boundaries, the interviewees first presented “women politicians” as an exclusive minority within their gender group. Second, they constructed intergroup categorizations by comparing women who meet the requirements to enter politics versus women who do not. When talking about identity contents, the interviewees constructed intergroup categorizations along the ideological axis only. Thus, they overlooked the differences between men and women who share the same ideology while they enhanced the differences among women of different ideologies. Overall, the interviewees constructed their “women politicians” identity as a subordinate identity within their overarching ideological identity rather than as a real intersectional identity. These results are discussed also in terms of discursive de-politicization of the “women politicians” intersectional identity.

This paper aims at examining how female politicians in charge of local political offices discursively construct their unique social identity as “women politicians.” We start from the idea that this identity stems from the intersection between female politicians’ gender and their ideological memberships. We will analyse how female politicians discursively construct their intersectional identity, focussing on how they manage and make sense of its multiple and potentially conflicting aspects. In this investigation, we start from a view of social identity as a social product that arises from group members’ discursive interactions (Reicher & Hopkins, 2001). According to this view, social identity can be studied as it emerges in discourses when group members dispute the boundaries and contents of their group membership—that is, when they construct and negotiate through their discourse who are part of the “we”, what makes “us” special and better, what “we” should do, and what “we” want to achieve in the political arena (Stuart, Thomas, Donaghue, & Russell, 2013).

In our analysis, we will pay special attention also to how female politicians discursively manage the politicization of their intersectional identity. The development of a politicized identity is a necessary condition for members of a disadvantaged group to mobilize collectively against discrimination (Kelly & Breinlinger, 1996; Klandermans, 2014; Simon & Klandermans, 2001; Van Stekelenburg, 2013). To our knowledge, how an intersectional identity may become politicized has been investigated only recently (Banfield & Dovidio, 2013; Klandermans, Van der Toorn, & Van Stekelenburg, 2008; Simon, 2011; Simon, Reichert, Schaefer, Bachmann, & Renger, 2015). Moreover, how politicization of an intersectional identity is constructed through group members’ discourses has never been analysed. Politicized identity does not imply only a consensus upon group norms, interests, and goals. It implies also shared adversarial attributions of the group disadvantaged condition and shared awareness that the collective disadvantage needs to be challenged in the public arena (Simon & Klandermans, 2001). So, we will analyse how discursive constructions of boundaries and contents of “women politicians” intersectional identity are related to discourses about gender discrimination in politics and to discourses about female politicians’ competence.

## Negotiating the Boundaries of Intersectional Identities

As regards group boundaries, the threshold for entering the in-group may be constructed either as low and soft, if the speakers’ aim is to include more and more people in the in-group, or as prescriptive and impermeable, if their aim is to construe a distinct and oppositional identity (Stuart et al., 2013). In the context of women’s under-representation in politics, how female politicians discursively construct the thresholds for being admitted among the “women politicians” might be linked to how they explain current gender discrimination in politics. Indeed, the way those thresholds are constructed in discourse might either challenge or legitimise women’s under-representation in politics.

Santos and Amancio (2016) analysed semi-structured interviews of Portuguese male and female politicians and examined how they talked about gender inequalities in politics. They observed that both male and female interviewees “naturalized” gender inequalities in politics, that is, they attributed them to society and to history, and gendered the issue of merit. That is, instead of blaming male gatekeeping practices or biased media representations of women, they blamed the women themselves, who, according to the interviewees, should display more interest and commitment in politics. The gendering of merit along with the naturalization of gender inequalities combined to determine an overall disengagement from the issue of social change. As a result, in the interviewees’ discourses, the social problem of gender discrimination in politics was transformed into an individual-level problem, where mainly the victims are deemed responsible for their condition. Indeed, both male and female interviewees disapproved gender quotas and instead supported the principle of merit. The principle of merit was also closely linked with discourses about women’s competencies, both in terms of women’s failure to attain the competencies necessary in politics and in terms of doubt or mistrust.

Discursive construction of women’s competence in politics is key to the construction of female politicians as effective political agents. However, the issue of competence is much more delicate to manage for female politicians than for male ones. Probably as a result of traditional socialisation, female politicians are less likely to self-perceive as qualified for a public office than are males (Burrell, 1998). At the same time, when they decide whether to enter politics or not, women rely on their competence more than do men, as a means to gain legitimisation both in front of male colleagues and among the electorate (Fox & Lawless, 2004). As a result, female politicians’ discourses about who is admitted within the in-group of “women politicians” and who is not are very likely to focus on competence.

Discursive construction of women’s competence can be also potentially problematic for its incongruence with conventional stereotypes regarding femininity. On the one hand, women are confronted with widespread gender stereotypes whereby women are associated more with communal traits (warmth, empathy, and sensitivity) than with agentic traits (strength and dominance). As a consequence, their ability to act as effective leaders, who are commonly stereotyped as agentic and dominant, is questioned. On the other hand, women who display agentic, stereotypically masculine traits are often depicted as cold and distant (Bligh, Schlehofer, Casad, & Gaffney, 2012). As a consequence, for example, discursive construction of women’s ambition is delicate. Female personal ambition can be more acceptable either if its beneficiary is said to be the nation rather than oneself, or if ambition is combined in discourse with communal traits (Hall & Donaghue, 2012). Even female politicians who are prime ministers construct for themselves a non-ambitious identity (Sorrentino & Augoustinos, 2016). Studies on how women nominated to public offices, for example as ministers or prime ministers, are discursively represented in the media show that women are trapped in a double bind. Actually, either media representations question women’s capacity of carrying out their political duties, as they are depicted as conflicting with their motherhood obligations, or they represent women who display full commitment to their political duties as evil mothers who overlook their family’s well-being (Garcia-Blanco & Wahl-Jorgensen, 2012; Verge & Pastor, 2018). Moreover, even when female politicians are unmarried and child-free, this lack of family can be imputed against them as a reason for their inability to get in touch with ordinary families (Sorrentino & Augoustinos, 2016).

Discursive constructions of gender discrimination in politics and of female politicians’ competence might be relevant to the discursive politicization of the “women politicians” intersectional identity. Actually, the construction of a disadvantage as collective and blameable upon a powerful out-group contributes to identity politicization (Iyer & Ryan, 2009; Simon & Klandermans, 2001). Interestingly, when members of a disadvantaged group do not perceive their collective condition as unfair discrimination perpetrated by a more powerful out-group, identity politicization is likely to be deterred (Simon et al., 2015). Indeed, perceived group discrimination leads to increased politicized identity (Branscombe, Schmitt, & Harvey, 1999). For example, women in counter-stereotypic occupations show augmented gender identification when they recognize gender-based disadvantages (Redersdorff, Martinot, & Branscombe, 2004). Also the discursive construction of female politicians’ competence might be relevant when examining how the intersectional identity of “women politicians” is constructed and politicized. Indeed, discourses about female politicians’ competence can speak to the capacity of female politicians as a group to collectively challenge gender discrimination in politics (Mummendey, Kessler, Klink, & Mielke, 1999; Van Zomeren, Spears, Fischer, & Leach, 2004).

## Negotiating the Content of Intersectional Identities

As hinted above, through their discourse group members also construct the contents of their identity, that is, “our” distinctive features, “our” interests and goals, what “we” should do and what “we” pursue collectively. In the realm of political activism, activists often face the challenge of having to manage and negotiate potentially conflicting aspects that stem from their multiple identities (e.g., Antaki, Condor, & Levine, 1996; Hopkins, 2011; Jaspal & Cinnirella, 2010; Stuart et al., 2013). In this regard, female politicians may experience peculiar complexities linked to their intersectional identity that result from their simultaneous gender and ideological memberships. Studies on intersectional identities have shown that people vary in their focus on intragroup similarities that arise from a shared identity and in their sensitivity to intragroup differences that spring from multiple memberships. As a consequence, individuals may recognize that their identity is rooted in multiple grounds (e.g., Black women may experience both their race and gender memberships as meaningful), take multiple axes of intergroup categorization as the source of their collective condition (e.g., gender and race), and develop their collective goals consistently. Alternatively, they may construct their identity on a single axis (e.g., Black women may experience just their race membership as meaningful) and develop the belief that social change can be pursued by targeting that axis, solely or primarily (e.g., race; Crenshaw, 1991; Greenwood, 2008). Similarly, one could explore how female politicians discursively construct their intersectional identity of “women politicians”, analysing how they base it on their gender and ideological memberships.

Ideological membership is likely to pose a divide in the discursive management of intersectional identities. Actually, ideological membership implies the endorsement of values that are perceived as absolute and non-negotiable principles. Indeed, values are represented in discursive interactions as very different along the ideological axis (e.g., Milesi, 2016; Sani & Reicher, 1998; Tetlock, Kirstel, Elson, Green, & Lerner, 2000). This is relevant to the discursive politicization of the “women politicians” intersectional identity. Actually, perceived similarity of values within the in-group is an important condition for individuals to construct themselves as a politicized group. The process of identity politicization implies that, thanks also to intragroup communication, individuals’ values and moral motivations are transformed into group concerns (Alberici & Milesi, 2016, 2018; Milesi & Alberici, 2018; Turner-Zwinkels, Van Zomeren, & Postmes, 2015; Van Zomeren, 2015; Van Zomeren, Postmes, & Spears, 2012). Indeed, when political activists perceive a basic diversity and heterogeneity in core values within their group, their perception of the group as an entity decreases, as does their identification with the group itself, which can even result in group schism (Sani, 2005). As a consequence, the discursive construction of the “women politicians” intersectional identity might be especially challenging for female politicians as they have to manage their gender similarity and simultaneous ideological diversity with women of different political groups.

## Aims

Following other researchers who have adopted the intersectional perspective to social identity (e.g., Greenwood, 2008; Hopkins & Greenwood, 2013), we assumed that interview data could provide insight into how female political officers experienced and made sense of their unique identity that resulted from the intersection between their gender and ideological memberships. Actually, the literature on the discursive construction and management of multiple social identities has acknowledged the value of qualitative methods (Antaki et al., 1996; Hopkins, 2011; Linden & Klandermans, 2007; Stuart et al., 2013).

Based on this, we interviewed women who had been elected in the lists of right-wing and left-wing political parties in local elections and were currently in charge of political offices. Right-wing parties favoured policies that support free market economy, law and order, and traditional families. In contrast, left-wing parties advanced policies in favour of equal opportunities, civil liberties, and tolerance for diversity. Consistently, supporters of right-wing parties endorse conservation and self-enhancement values (e.g., tradition and success) while supporters of left-wing parties endorse self-transcendence values (e.g., universalism and benevolence; Caprara, Schwartz, Capanna, Vecchione, & Barbaranelli, 2006).

We examined the interviews by focussing on how the interviewees discursively constructed their intersectional identity of “women politicians” and on how they managed its multiple and potentially conflicting aspects. Within this overarching theme, we explored: 1) how the interviewees constructed the boundaries of their intersectional identity, taking into account also their discourses about gender discrimination in politics and about female politicians’ competence; 2) how they constructed the content of the “women politicians” identity, focussing our attention also on the interviewees’ discourses about their own and other women’s values.

## The Research Context

In Italy, female politicians are still a minority. On the whole, according to the Global Gender Gap Index 2017, Italy is in 82nd position out of 144 countries examined in terms of gender equality.^i^ And the situation is even more worrying for what concerns women representation in politics. At the level of national politics, none of the main political parties is led by a woman, and the gap between men and women widened in 2017 compared to previous years; in 2016 Italy was in 25th place, and in 2017 it went down to 46th. This result seems also in contrast with the recent European trend; 2017 elections held throughout European countries increased parliamentary representation of women up to 27.1 per cent, up from 26.3 per cent in 2016. Within the European region, however, Italy’s overall performance in terms of gender equality in politics is rather negative, as it is placed among the last positions among the European countries, above only Cyprus, Portugal, Romania, Croatia, and Slovakia.^ii^ The 2018 Italian national elections were carried out by applying a recent electoral law that states that at least 40 per cent of the candidates must be women. Despite this, in all major parties the percentage of women elected was around 30 per cent, significantly below the minimum threshold of 40 per cent. This is probably a consequence of how Italian major parties have chosen women candidates, versus men. For example, it is possible that men were more frequently nominated as candidates within those electoral colleges where election was guaranteed, making it more difficult for women to have a chance to be elected.

At the level of local politics, women are slightly better represented, especially in small to medium municipalities. Currently, the percentage of women who are seated in municipal councils is 39 per cent in municipalities with fewer than 15.000 inhabitants and 40 per cent in municipalities with more than 15.000 inhabitants. However, in capital cities, the percentage of women who are seated in municipal councils is 22.2 per cent. Overall, women mayors are only 14.1 per cent of the total. As regards the territorial distribution, women representation is greater in the north of Italy, with 30.6 per cent in the north-west and 31.7 per cent in the north-east, while in the centre it is 29.7 per cent and in the south and in the islands it is 26.8 per cent. As regards the distribution of political offices, there is a clear territorial gap with respect to the number of mayors, since north-west female mayors represent almost half of the overall number of Italian mayors (46.5 per cent), and a similar percentage has been shown for female deputy mayors. Albeit a minority, women involved in politics are much more educated than their male counterparts. For women involved in local politics, 46.2 per cent have a degree or a PhD, versus 31.6 per cent of men. For men who are involved in local politics, 21.1 per cent have barely completed compulsory education, compared to 9.8 per cent for women (ANCI, 2017).^iii^

## Method

### Participants and Interviews

The present research was carried out in Italy in 2015. At the time of the interviews, all interviewees held a political position at the local level, in particular in the municipalities or the provinces of north-west Italy. As reported above, this area of the Italian territory is characterized by having a higher presence of female administrators than other Italian areas. Thus, interviewees developed their political career in one of the most favourable contexts in Italy. The interviewer initially contacted 15 women personally, and 10 of these agreed to be interviewed. The sample is both small and unrepresentative, and we do not make any generalizations from our findings to other Italian female politicians. Participants were informed that their anonymity would be preserved and that their privacy rights would be strictly observed so that it would not be possible to identify them from the results. All interviews were held at the offices of female politicians and were audiotaped and then transcribed verbatim.^iv^ Non-linguistic features of speech (e.g., pause lengths and stress) were irrelevant for the purpose of the present paper and thus were not transcribed. Interview lengths ranged from 39 to 113 minutes (average of 77 minutes). Interviewees were also informed that they could ask to switch off the audiotape or to withdraw immediately from the research at any time and that, if this were the case, their answers would not be considered. Moreover, participants were assured that their answers would be used for research purposes only. No participant withdrew during the interview. After the interviews, all participants were fully debriefed about the aim of the research and offered to receive the research final results.

As reported in Table 1, five interviewees belonged to centre-right parties and five to centre-left ones. Participants’ ages ranged from 35 to 59 years, and their political experience ranged from 8 to 40 years of previous political activism. Seven interviewees reported having a degree, while three received an upper secondary education. All interviewees were married/cohabitating, and only two of them did not have children.

**Table 1:** Details about the Interviewees

Interviewee	Political role	Political orientation	Age	Marital status/marital cohabitating	Number of children	Political activism length	Education
1	Municipal councillor	Centre-left	52	yes	-	35 years	Master’s degree
2	Province councillor	Centre-right	35	yes	1	15 years	Master’s degree
3	Province vice-president	Centre-left	59	yes	2	40 years	Master’s degree
4	Municipal councillor	Centre-right	46	yes	-	8 years	Master’s degree
5	Municipal councillor	Centre-right	58	yes	1	39 years	Master’s degree
6	Municipal council vice-president	Centre-right	53	yes	1	17 years	Upper secondary education
7	Municipal councillor	Centre-right	47	yes	1	9 years	Upper secondary education
8	Deputy mayor	Centre-left	47	yes	1	30 years	Upper secondary education
9	Province councillor	Centre-left	41	yes	2	26 years	Master’s degree
10	Province councillor	Centre-left	52	yes	1	35 years	Master’s degree

### Interview Schedule

Through semi-structured interviews, participants were asked to talk about their political careers and to reflect on the condition of women in politics. The topics included the initial decision of the interviewees to engage in political activity (*How did you get involved in politics?*), to enter political competition (*Could you describe how you started your political career?*); their perceptions of parental and familiar support (*What does your family think about your political activity?*); their understandings of political activism, their political careers, and the costs and benefits of being a politician (*What does politics mean to you? What does it mean to be a politician?*); their perceived ties with both other women and men politicians within their own and other parties (*What is your attitude toward your political colleagues, females and males, both of your and other parties?*); their explanations for the under-representation of women in Italian politics (*How do you explain female under-representation in Italian politics?*). The interviewer relied on an interview guide that contained the key questions reported above but it also oriented questions and cues according to participants’ response.

### Analytic Strategy

We followed other research based on the social identity approach that examined speeches and texts in order to explore how social identities and category relations are actively constructed and understood through discourse in the political arena (Hopkins & Greenwood, 2013; Pehrson, Stevenson, Muldoon, & Reicher, 2014; Rooyackers & Verkuyten, 2015). More specifically, we explored how the interviewees discursively managed their intersectional identity of “women politicians” and its potentially conflicting aspects (e.g., their position as an under-represented category, the similarities and differences within this category that stem from the intersection between gender and ideology). Our analytical approach was focussed on how participants accounted for their experience of “women politicians” in terms of the identities they saw as relevant. Our investigation was informed by the developments in research on intersectional identity and identity politicization reviewed above. We interpreted interviewees’ accounts as reflecting the experiences that we assume they have had during their political career and, above all, as reflecting the meaning that they attached to them. In doing this, we also assumed that the social categories the interviewees referred to in their discourse should be intended as the outcome of how participants interpreted and made sense of their experience, and as a consequence as fluid and negotiable.

Our analysis unfolded through three stages (Pehrson et al., 2014; Rooyackers & Verkuyten, 2015). First, both the researchers read the transcripts several times. The sections of the interviews that touched on the issues regarding women in politics, what it meant for the interviewees to be “women politicians,” what had been required from them to become so, and how they related with other women in politics were spotted and highlighted within the transcripts. Second, these sections were closely analysed with special attention to how the interviewees constructed the boundaries of the “women politicians” category as well as its contents. Within the topic of category boundaries, we focussed on how the interviewees talked also about women’s under-representation in politics and about female politicians’ competence. Within the topic of category contents, we focussed on how the interviewees constructed similarities and differences among “women politicians.” Finally, within each of these interrelated topics, we illustrated the patterns of meaning that the interviewees attached to the “women politicians” intersectional identity and to their experience of it.

## Analysis

### An Exclusive Intersectional Identity

First of all, we explored how the interviewees discursively constructed their intersectional identity of “women politicians” by focussing on how they constructed its boundaries, that is, the thresholds for being admitted to the category. Moreover, we explored how interviewees’ discourses about this issue were linked to how they constructed and explained women’s under-representation in politics and to how they talked about female politicians’ competence as a means to collectively challenge gender discrimination in politics.

The intersectional identity of “women politicians” is constructed as highly exclusive. Interviewees often highlighted that only a restricted minority of exceptional women are “admitted” in politics. For example, Paola^v^ (interviewee 8), a centre-left deputy mayor of a small town, believed that the group of “women politicians” includes “frontrunners” only, that is, special women who are better than other women.

**Extract 1**

Paola (interviewee 8): Unfortunately, women who enter politics are either immediately successful or they burn out, lose their way, and quit. […] Women have smaller margins for error than men do. If they are not frontrunners, they are relegated to secondary positions.

As we can see in Extract 1, the process whereby women are admitted in politics is constructed as a sort of a natural selection process. This process is presented as a descriptive norm that descends from the widespread gender discrimination in politics. Such a discursive construction may go to the extreme and close the “women politicians” category to potential newcomers. This happened when the interviewees transformed the descriptive norm of how women politicians are into the prescriptive norm of how women politicians should be. For example, when Caterina (interviewee 6), the municipal council vice-president of a centre-right party, called for more power for female politicians, she retained stricter criteria for women to enter politics than for men to do so.

**Extract 2**

Caterina (interviewee 6): I think that some laws should be written first by women only and later revised by both women and men. We must not leave some issues in men’s hands only. […] I believe that women should play a more important role in the country’s political life. I hope and fight for this. But these women should be worthy and prepared to win and gain power by being really better than men.

As a result, intergroup categorization shifts from “women versus men” to “women who meet the requirements to enter politics versus women who do not.” We can see such a shift in Extract 3, where Camilla (interviewee 4), a centre-right councillor in a small municipality, established strict criteria for admitting new members in the group of women politicians:

**Extract 3**

Camilla (interviewee 4): Women elected in Parliament achieved such positions because they had an edge over the others and because they were better than other women. We must send a message to Italian women, a message that says that although everybody can do politics, there must be something that elevates you above other women, that makes you special and better, because politics is a serious thing that needs skill and patience, passion and dedication, and the time and the desire to test oneself.

### Exclusiveness as a Result of a Stable Hostile Context

When interviewees talked about the exclusiveness of the “women politicians” category, they often explained it with reference to the toughness of the political context. Indeed, according to all interviewees, gender discrimination in politics is widespread; interviewees unanimously agreed that there are deep prejudices and hostile behaviour toward female politicians. In Extract 4, Matilde (interviewee 7), the municipal councillor of a centre-right party, was explicit in saying that female politicians are targets of male prejudice and discrimination, and she voiced her negative affective reactions about it. At the same time, she constructed discrimination against women as a stable feature of politics.

**Extract 4**

Matilde (interviewee 7): I believe that unfortunately politics is still a male chauvinist environment, where men join together to make fun of women and to exclude them from power. […] In my opinion, the obstacles that we as women encounter when striving for public offices are due to the male chauvinist traits of politics, traits that can be found across parties. […] I realize that there is deep prejudice against me and against my women colleagues. We are women, we are newcomers, and there are always some critics of us. […] I have just realized this and I am furious; I mean it is frustrating and not reassuring.

Discourses on gender discrimination in politics were intertwined with discourses about the traditional gender roles the interviewees are required to play outside politics. For example, when the interviewees focussed on female politicians’ underinvestment in social capital, they explained it with reference to the traditional care role they play in the family. While no interviewee discussed the stability of those traditional gender roles, all of them emphasized that it was really very hard for them to find a balance among their different roles in the family, at work, and in politics. According to interviewees’ discourses, they sought a balance between these different roles, which leaves women with so little time to socialize with male colleagues that it is almost impossible for them to build the social networks that are necessary for political success. For example, in Extract 5, Angela (interviewee 10), a centre-left province councillor, attributed women’s under-representation in politics to their underinvestment in social capital due to the heavy family burdens that women in politics have to bear.

**Extract 5**

Interviewer: How do you explain the low percentage of female politicians in Italy?

Angela (interviewee 10): I think that, when a woman chooses to do active politics, she is aware of the toughness of the context, and she is aware that this will bring about family problems. It is paradoxical that men are requested to develop gender politics in favour of women when politics is organized as a function of male time. Conciliation is the greatest problem. […] Men are much more sponsored; they are enabled to build social networks and social relationships that are much wider than those of women. Actually, contrary to men, women do not have time to hang around with friends and to meet new people.

So while the interviewees acknowledged gender discrimination in politics, at the same time they attributed it to societal features that were described as stable and really unlikely to change or to be challenged.

### Exclusiveness as a Result of High Standards of Competence

The interviewees constructed politics as an environment that generally is hostile to women because it sets very selective thresholds for women to enter. Interviewees’ discourses often focussed on competence as a necessary requirement for women to enter politics. Moreover, when interviewees talked about who is admitted in the “women politicians” category and who is not, they highlighted that there are special criteria that women are required to meet while men are not. Thus, competence is constructed as a source of discrimination insofar as it is presented as checked and required from women but not from men. Consequently, exclusiveness of the “women politicians” category is attributed to the fact that women are required to show a level of competence that men are not. Interviewees often stressed that they, as women, are confronted with continuous competence tests. In this regard, as we can see in Extract 6, Matilde emphasized that women politicians are constantly required to show they are capable and suitable for their offices, while men are not.

**Extract 6**

Matilde (interviewee 7): We are asked to meet requirements that nobody would dream to ask of men. […] I believe that things such as competence and expertise are required to women as a pretext to slam the door in their faces. […] I believe that women in politics are so few because men do not want them and act in a way so as to create a number of obstacles that are impossible for women to overcome. […] It is painful to think that for all her life long, along all her political activity, a woman has to keep showing that she is suitable for her office, while I clearly perceive that this is not required of a man.

Quite paradoxically, interviewees often presented themselves, as competent and successful as they are, as an exceptional, fortunate case compared to other women. In Extract 7, Camilla stated that she represented a rare and fortunate case, and she attributed her success not so much to her competence as to an external factor, that is, her good luck to encounter people who trusted her—as well as to her decision not to have children.

**Extract 7**

Camilla (interviewee 4): I have to admit to myself that, unfortunately, not all women had my fortune, that is to have a chance in politics. I had someone who believed in me, and I was so fortunate of being able to manage my public and private life. […] I can also say that I have the fortune or misfortune not to have children, which is one problem less.

Competence was prevailingly presented as the technical and sectorial knowledge that the interviewees achieved thanks to their individual hard work and study. Only in a few isolated cases did the interviewees report that their party provided them with relevant training opportunities when they started their public activities. Quite the contrary, most interviewees said that they attained their competencies through huge individual effort and by overcoming a number of situational obstacles, often represented by duties of care for young children and/or elderly parents. As a result, interviewees constructed their personal competence as an instrument that they individually achieved thanks to great personal sacrifices rather than as a means that other female politicians are likely to possess and that female politicians as a group could employ to achieve gender goals.

So discursive construction of female politicians’ competence is based not only upon the intergroup categorization “women versus men” but also, and sometimes primarily, upon the intergroup categorization “women who have the competence required to enter politics versus women who do not.” “Women politicians” are often constructed as exceptionally competent and as exceptionally lucky; in contrast, women who do not enter politics are depicted as unable and/or unwilling to make the sacrifices necessary to achieve the level of competence that is required from female politicians. In either case, the outcome of the interviewees’ discourses is that “women politicians” were described as exceptional cases within their gender group.

Moreover, the interviewees focussed on the issue of competence in a way that mirrors the tension between women’s widespread self-perception of being not qualified enough for public office, compared with men, and one’s own personal hard work to gain competence as a means of legitimisation. Quite often the interviewees associated female politicians’ competence with anxiety, represented as a (stereotypical) stable female feature that men do not have. Such a feature is sometimes constructed as a sort of an extreme conscientiousness that drives female politicians to strengthen their credentials and eventually to make them even more competent than men. In most cases, however, it was presented as a drawback, a basic female lack of self-confidence that impedes the success of most women in politics. For example, in Extract 8, Francesca (interviewee 3), the centre-left province vice-president, argued that women often doubt their political competence because they have greater anxiety and insecurity than men.

**Extract 8**

Francesca (interviewee 3): Politics makes women worry much more than men. I often ask myself whether I am able to make it; men are different. As women, we always question ourselves about our abilities, and we are much more anxious than men, and sometimes this has negative consequences.

To summarize, interviewees constructed the boundaries of the “women politicians” intersectional identity as highly prescriptive and impermeable, generating a distinct, minority oppositional identity. Moreover, the intersectional identity of “women politicians” was contrasted not only against men in politics but also, and sometimes primarily, against women who are not engaged in active politics. As a result, this discursive construction of category boundaries sounded like a legitimisation of women’s under-representation in politics as, by definition, exceptional women are a minority.

### The Gender Ingroup as Affected by Deep Ideological Differences

As to the contents of the intersectional identity of “women politicians”, we explored how the interviewees discursively managed the potentially conflicting aspects stemming from their gender similarity and simultaneous ideological diversity, as compared with women of different political groups.

Generally speaking, ideological membership emerged as the main axis along which the interviewees accounted for their experience as “women politicians”. As a consequence, in most cases, the interviewees managed the conflicting aspects of their intersectional identity by emphasizing the ideological differences among female politicians. The complex intersection between gender and political memberships is clearly constructed with the aim of showing one’s deep loyalty to core cherished values, above and beyond gender similarities. Emphasis on ideological membership brought to the foreground different and often opposed political goals among women politicians. Lucia (interviewee 9), a centre-left province councillor, was explicit in saying that female solidarity and unity among female politicians of different parties is very unlikely, since differences among them in core values are too deep. Interestingly, hypothetical solidarity towards another member of the same gender group but different ideological group was constructed as a bias.

**Extract 9**

Lucia (interviewee 9): I do not think there’s a female solidarity across different parties. […] We basically refer to core values, and I do not believe I can share values and worldviews with those who made choices that are radically opposed to mine, even if they are women. […] I do not allow my political judgment to be biased, for example, by the mayor being a man or a woman. With the former centre-right mayor, who was a woman, there was neither common ground or shared values because our political ideals were profoundly different.

Some shared gender goals were constructed when the interviewees referred to the specific contexts of boards for equal opportunities. Interviewees who worked for gender equality in local boards reported cooperation among women of different parties and ideologies. Under those specific circumstances, just for the sake of women’s interests, they stated that they overlooked ideological divisions. The interviewees, however, constructed such a collaboration as an exceptional and restricted condition. As can be seen in Extract 10, Angela combined the description of sharing values with women of different political parties with an emphatic reference to the ideological differences that otherwise divide them, so that ideology rather than gender emerges as the main axis for intergroup categorization.

**Extract 10**

Angela (interviewee 10): It is becoming more and more difficult for women in politics to find a common ground because party interests are very strong. Maybe, at the local level, like a province, an allegiance for women interests can be made, but at the national level it is much more difficult because parties have to show their strength. I have met centre-right women who share some ideals and values with me and also the desire to make Italy better and fairer.

Moreover, it is worth noting that the interventions promoted by local boards for gender equality aimed mainly at improving the condition of working women in general (e.g., proposals of change in the opening and closing times of nursery schools or proposals of support to families with children with physical or psychological disabilities). In no cases did the boards deal with the specific disadvantaged condition of female politicians.

Differences within the interviewees’ gender group were often constructed also at the level of interpersonal relations. Indeed, the interviewees constructed interpersonal conflicts among women as much more divisive than conflicts among men, and they explained this divisive female attitude by referring to a stable feature of the female gender. The interviewees described the category of “women politicians” as affected by deep inner conflicts around the achievement of powerful positions. Women politicians were presented to be much more inclined to fight against each other than to act in a united way and to provide reciprocal gender solidarity. According to the interviewees’ accounts, women politicians would prioritize their individual interests over gender ones, unlike male politicians, and competition for power among women would be stronger than among men. This account hindered a discursive construction of shared collective gender goals. In this regard, Caterina reported:

**Extract 11**

Caterina (interviewee 6): I see when I go to municipal councils to discuss with women councillors belonging both to centre-left majority and to my own political party, there is no agreement. For several reasons, both political reasons but also human reasons. In short, while men never put forward dislike or envy as reasons for not to vote for a fellow, women do so.

### The Ideological Ingroup as Providing Support to Women

Consistent with constructing their experiences as a function of the ideological axis, when the interviewees talked about their ideological membership, they discounted gender differences. As a consequence, the interviewees constructed their own party as rid of biases against women’s political career. Actually, most interviewees were proud to say that their own parties had provided them with a chance for political careers and that their male party colleagues were supportive of rather than prejudiced against women. Paola and Camilla, for example, compared the context of politics in general, where the relationships between female politicians and their male counterparts are hostile, with the specific context of their own party, where they said they had found wide support from their male colleagues.

**Extract 12**

Paola (interviewee 8): I feel myself as a “woman politician”, and I have had to build my own defences, but for years I have had a party that has made me grow and that has protected me. […] My colleagues, even if mostly men, chose me as mayoral candidate and I accepted it. […] When someone has the potential to run for a political office, even if she is a woman, the party does not boycott her.

**Extract 13**

Camilla (interviewee 4): In my party, the atmosphere is completely different. Women are much considered and not just because of gender quotas but because the party wants to acknowledge the role they deserve. It is a message that as women play a key role in the family, they can play a similar role also in civil society.

So it is within the discursive framework of one’s own ideological membership that the identity of “women politicians” is provided with a meaning. Being nested in ideological membership, the “women politicians” identity is discursively managed more as a subordinate identity rather than as a real intersectional identity. Moreover, such a favourable description of one’s own party is functional to protect the in-group from the overall negative evaluation of politics as prejudiced against women. However, at the same time, it conveys that gender discrimination is not so widespread, thus making it a problem of other parties rather than one’s own.

Only in the case of right-wing interviewees did gender become the main axis along which the interviewees accounted for their experience. This is consistent with the high cognitive centrality of gender categorization in right-wing people (Peterson & Zurbriggen, 2010; Schreiber, 2002, 2008). For example, Elisabetta (interviewee 5), a centre-right municipal council councillor, employed the “women versus men” intergroup categorization to account for all her experiences across various life domains: from family, where male members are not expected to help their female counterparts, to politics, where male colleagues are ready to take advantage of women. She stated that gender equality had consistently been at the top of her political agenda within the local board and, as we can see in Extract 14, she wished that, sooner or later, women politicians as a group could subvert their disadvantaged condition.

**Extract 14**

Elisabetta (interviewee 5): When meetings for the municipal council began at 7 p.m., my husband and my son arranged for dinner. Of course, I cooked something and then they arranged something else. It was a matter of arranging: I do not expect any help from men. […] Women are much stronger than men. I mean, men cannot bear the rhythm of women. […] Women in politics must show that they are stronger than men, since, otherwise, in three minutes men trample on you and you stay blocked in the kitchen. […] There are only a few women in politics because only a few of them believe in themselves. In my opinion, women have terrific potential but, as I said before, there are women who let men prevaricate. Why do they accept subordination? It is not fair. I know that, if we wanted, we could really make a cultural revolution.

In Elizabetta’s words, two ingredients of discursive politicization of the intersectional “women politicians” identity can be found: the reference to gender discrimination in politics, and the reference to women politicians’ strength and potential. However, it is not only commonality of values and goals among “women politicians” that is still lacking. Gender discrimination is also attributed internally to women’s lack of self-confidence. So, although Elisabetta wished for a “cultural revolution”, her wish was embedded in a discursive context where no real politicized intersectional identity was constructed. Other right-wing interviewees mentioned some women-only training courses that aimed to promote the careers of women politicians, but these courses were embedded within the party boundaries and never involved women of different ideologies.

## Discussion and Conclusion

This paper explored how female politicians discursively construct their intersectional identity as “women politicians” (Greenwood, 2008). With this aim, we interviewed female political officers and examined how they talked about the boundaries and contents of their “women politicians” identity (e.g., Reicher & Hopkins, 2001). We also analysed how interviewees’ discursive constructions of the “women politicians” intersectional identity were related to their discourses about gender discrimination in politics and to their discourses about women politicians’ competence. As a consequence, our results can also speak to the discursive politicization (or depoliticization) of the “women politicians” intersectional identity (Simon & Klandermans, 2001; Simon et al., 2015). Our analysis reveals the complexities of the interviewees’ discursive management of their intersectional identity as well as its discursive (de)politicization.

When talking about identity boundaries, first, the interviewees constructed “women politicians” as an exclusive minority within their gender group, a restricted elite among ordinary women. Actually, the thresholds to be admitted to this group are constructed as so demanding that only a minority of women are allowed to enter. Indeed, the interviewees constructed intergroup categorization along the axis of “women who meet the requirements to enter politics versus women who do not” rather than along the gender axis of “women versus men”. Those discursive constructions of intergroup categorizations were embedded in a description of politics overall as hostile to women. As a result, interviewees’ discourses sounded like a justification of women’s under-representation in politics rather than like a challenge to their collective disadvantaged condition. The discursive justification of women’s under-representation in politics was accompanied by descriptions of male gatekeeping practices and traditional gender roles as stable features of politics (Francescato, Mebane, Sorace, Giacomantonio, & Lauriola, 2008).

Second, the interviewees constructed the boundaries of the “women politicians” identity also by referring to the continuous tests of competence with which women politicians are confronted. The interviewees constructed it as a source of discrimination, as women would be required to show their suitability for office throughout their career while men would not be. However, the interviewees constructed their level of competence, and the process whereby they achieved it, as so exceptional within their gender group that once again the intergroup categorization was constructed along the axis “women who meet the requirements to enter politics versus women who do not” rather than along the “women versus men” gender axis. Moreover, although the interviewees presented themselves as exceptionally competent among women, they generalized the basic lack of self-confidence to their whole gender group, thus mirroring the stereotypical belief that women would not be up to the task of political competition (Fox & Lawless, 2004). Finally, by focussing on the heavy personal sacrifices the interviewees made to achieve competence, the interviewees constructed a distance between themselves as successful women in politics and other (less successful) members of their gender group. This discursive construction is consistent with the finding that the so-called “queen bees”, that is, women who are successful in male-dominated contexts, underline dissimilarities and distance themselves from other women who have been less successful (Derks, Van Laar, & Ellemers, 2016).

Third, when interviewees talked about the contents of their intersectional identity as “women politicians” (i.e., what makes “us” special and better, what “we” should do, and what “we” want to achieve), they did not construct intergroup categorization along the gender axis, but rather along the ideological one. Indeed, the differences between men and women who shared the same ideology were overlooked in interviewees’ discourses. Consistently, the interviewees’ own party was constructed as fully supportive towards women’s political careers. Instead, the differences among women of different ideologies were enhanced, often thanks to discursive reference to non-negotiable values. In the discursive construction of the “women politicians” intersectional identity, interviewees associated such deep meanings to their ideological identity that the differences in central beliefs and core values within the group of women in politics were emphasized more than the similarities. Discursive focus on conflicts among women is consistent with a widespread tendency to problematize same-sex conflict when it occurs between women much more than when it occurs between men, which deflects attention away from structural obstacles that women encounter and towards women’s alleged deficiencies, thus justifying women’s disadvantaged condition (Sheppard & Aquino, 2017). Consistently, cooperation among women was presented either as restricted episodes due to contextual aims (e.g., equal opportunities boards) if it involved interaction among women of different ideologies, or as part of the interviewees’ party activities if it involved women of the same ideology. Thus, being constructed as sporadic episodes or nested in interviewees’ ideological membership, the “women politicians” identity was managed discursively more as a subordinate identity within the interviewees’ ideological identity than as a real intersectional identity.

These results are also relevant in terms of discursive de(politicization) of intersectional identity. Actually, on the one hand, women’s under-representation in politics was acknowledged in interviewees’ discourses; on the other, however, it was justified rather than challenged, and no likelihood of social change was envisioned. While discursive acknowledgement of women’s under-representation in politics might have the potential to politicize the intersectional identity of “women politicians”, its discursive justification defuses such a potential. Furthermore, the challenges that the interviewees associated with their individual achievement of competence and with its public deployment impeded them from generalizing their own competence to the whole gender group, while they did generalize the stereotypical female lack of self-confidence. As a result, the intersectional identity of “women politicians” was constructed as a restricted category of extraordinary women. The exceptionalism that results from these discursive constructions of identity boundaries was inconsistent with discursive politicization of the “women politicians” intersectional identity.

The present study reveals the discursive complexities arising from the interviewees’ simultaneous gender and ideological memberships. Previous research on women’s under-representation in politics, especially in leadership positions, has found a labyrinth of obstacles that women encounter, from male gatekeeping practices to fewer opportunities for women to access the media and biased media representations of women, from difficult conciliation among family, work, and political roles to women’s underinvestment in social capital (e.g., Eagly & Carli, 2007; Francescato et al., 2008). Our research adds to these findings by suggesting that a further obstacle can be found in how female politicians understand and discursively construct their intersectional identity of “women politicians”, basically depoliticizing it.

Although our qualitative methodology does not allow us to establish causal relationships, these findings suggest that self-perceived exceptionalism could undermine a discursive construction of politicized identity, gender discrimination, and group efficacy that would be strategic to collectively challenge the status quo.

We acknowledge that this study is limited by the restricted sample and by its being focussed on a specific sociocultural context. Although female politicians constitute a minority that is hard to reach, we hope that future studies will examine larger samples of participants and will extend the investigation to different contexts. Findings obtained in the present research indeed deserve further investigation also through quantitative methods. Moreover, it would be interesting to enlarge the investigation to less successful female politicians in order to explore how they construct the intersectional identity of “women politicians” (e.g., whether they embed it within an overarching ideological identity or they construct intergroup categorization also along the gender axis) and whether and how they politicize it through discourse. Finally, we observed that in their discourses the interviewees widely used modal categories referring to “women politicians” (i.e., women politicians must/have to/can do…). Although an analysis of the use of modal verbs and modal expressions is out of the scope of the present research, we acknowledge that this could be a fruitful avenue for future research. Actually, the modal articulation contributes to shaping the positioning of the subject and his/her relationship with other actors in terms of duties, responsibilities, or degrees of free will, as well as with his/her sense of agency (De Luca Picione, Martino, & Freda, 2017, 2018).

At an applied level, our research suggests that group-level interventions are needed to nurture the intersectional identity of women politicians. Previous studies of female networking and women-only training opportunities in non-political organizations have shown that such practices, under given conditions, can play a social identity function (Hersby, Ryan, & Jetten, 2009; Kirton & Healy, 2004). Actually, female networking can provide women with a sense of belonging and a professional shared identity; women-only courses, by privileging women’s issues, can acknowledge perceived gender discrimination and, through shared learning, provide a sense of personal and collective efficacy, which might favour the politicization of the intersectional identity of women politicians. Indeed, it would be interesting to investigate whether and when these practices could play a similar function also in the political context, where, beyond gender membership, strong ideological identities are present.
